# Generation of TALEN-Mediated GR^dim^ Knock-In Rats by Homologous Recombination

**DOI:** 10.1371/journal.pone.0088146

**Published:** 2014-02-11

**Authors:** Verónica Ponce de León, Anne-Marie Mérillat, Laurent Tesson, Ignacio Anegón, Edith Hummler

**Affiliations:** 1 Department of Pharmacology and Toxicology, University of Lausanne, Lausanne, Switzerland; 2 INSERM UMR 1064 and Transgenic Rat Facility, University of Nantes, Nantes, France; Florida International University, United States of America

## Abstract

Transcription Activator-Like Effector Nucleases (TALEN) are potential tools for precise genome engineering of laboratory animals. We report the first targeted genomic integration in the rat using TALENs (Transcription Activator-Like Effector Nucleases) by homology-derived recombination (HDR). We assembled TALENs and designed a linear donor insert targeting a pA476T mutation in the rat Glucocorticoid Receptor (*Nr3c1*) namely GR^dim^, that prevents receptor homodimerization in the mouse. TALEN mRNA and linear double-stranded donor were microinjected into rat one-cell embryos. Overall, we observed targeted genomic modifications in 17% of the offspring, indicating high TALEN cutting efficiency in rat zygotes.

## Introduction

Customized nucleases (Zinc Finger Nucleases, TALENs and CRISPRs (Clustered Regularly Interspaced Short Palindromic Repeats) [1]–[4]) have revolutionized the genetic engineering field. TALENs are DNA binding nucleases that can be tailored to bind almost any sequence. It has been reported that a TALEN cleavage site can be found every 35 bp of genomic DNA [5]. TALENs are versatile molecules that can be easily designed, cloned, assembled and tested in a molecular biology laboratory. With the description [6], [7], and assembly [8], [9] of TALENs, the genetic engineering field has made important progress. Today, there are several web-accessible software available for the design and assembly of customized TALEN arrays. The most used are: TALE-NT or the newest version TALE-NT 2.0 [5], [10], developed by Voytas and Bogdanove, where TALENs are assembled using the golden gate strategy [11], and a standard cloning assembly named REAL (Restriction Enzyme And Ligation) [12] developed in Joung's laboratory, that includes high throughput techniques [13]. Gene disruption (knock-out) by TALENs has been achieved recently in several organisms including *C. elegans* [14], zebrafish [15], [16], mice [17], and rat [18], [19].

The rat has been historically a well suited model for health-related research fields such as physiology [20] and behavioral genetics [21]. However its use has been hampered due to the difficulty of culturing embryonic stem cells (ESC) and difficult gene manipulation techniques [22]. We report successful gene targeting (Knock-in, KI) in the rat by TALEN mRNA microinjection in one-cell embryos, which requires no ES cell culture and is efficient.

## Results

### TALEN design and assembly

**Figure 1:**
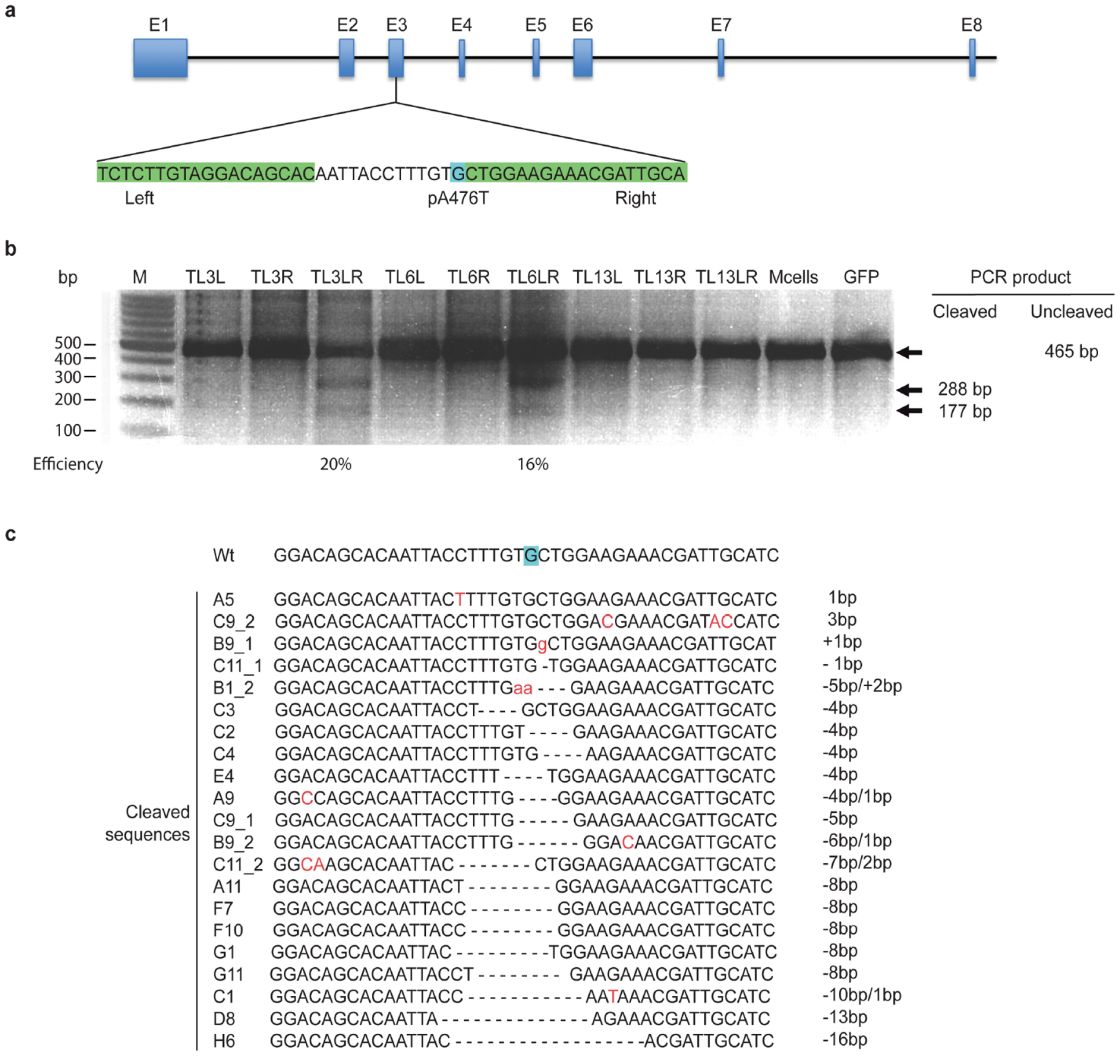
TALEN design and evaluation of cutting efficiency in rat glioma C6 cells. (a) Schematic of the rat *Nc3r1* (GR) gene. Zoom on the area of the mutation pA476T in exon 3. The first nucleotide of the 476 codon is highlighted in blue. TALEN binding sites of TAL 3 are highlighted in green. Detailed sequences of TAL 6 binding sites can be found in Table S1, File S1. (**b**) T-endo1 assay results. Pooled DNA from C6 cells transfected with either Right, Left or Right and Left TALEN monomers (marked with R, L or RL, respectively) was amplified and treated with T7 endo 1 enzyme. Cut bands of 288 and 177 bp indicate TALEN activity. Mcells are mock transfected cells, GFP: GFP transfected cells were used as a positive transfection control. Intensity of the cut bands are indicated for TAL 3 and TAL 6 pairs. (**c**) TAL 3 transfected cells screening. PCR amplicons of the region around the pA76T mutation were subcloned into TOPO vector. Clones were isolated and analyzed individualy. Four point mutations and insertion.s are marked in red.

Using the Zifit Targeter **(Materials and Methods)**, we obtained a set of 18 TALEN pairs using two query sequences indicated in **Table S1 in File S1**. All TALENs targeted the pA476T mutation in the rat glucocorticoid receptor (GR, *Nr3c1*) (**Fig. 1a**), corresponding to the pA458T mutation in mouse, that inhibits GR dimerization, namely GR^dim^ [23]. Following the guidelines for TALEN-site selection in Cermak et al. [5], we selected 3 TALENs: TAL 3, TAL 6 and TAL 13. We assembled the 3 TALENs into heterodimeric FokI expressing vectors described in [24] following the REAL and REAL-Fast standard-cloning assembly method from TALengineering.org (see **Table S1 in File S1** for detailed TALEN binding sequences).

### Functional analysis of TALENs in rat C6 glioma cells

We transfected rat glioma C6 cells with all three TALEN pairs and incubated them at 30°C during 72 hours. Western blot indicated that both right and left TALEN monomers of the TALEN pairs 3 and 6 were expressed (**Fig. S1 in File S1**). However, the right TALEN 13 monomer was not highly expressed in cells. We therefore proceeded with TALEN pairs 3 and 6 for further experiments. Prior to injection in rat zygotes, we assessed TALEN nuclease activity in C6 cells by a DNA mismatch detector assay: T7 endonuclease I (T7 endo I). We transfected C6 cells with either right or left TALEN monomers or both, or a GFP expressing plasmid for transfection efficiency control. After incubation for 72 hours, we harvested the cells and amplified genomic DNA to obtain amplicons of 465 base pairs (bp) around pA476 **(Materials and Methods)**. DNA amplicons were then denatured at high temperature and annealed to form heteroduplexes that were detected by the T7 endo I nuclease. Primers used are listed in **Table S2 in File S1**. Resulting bands were 177 and 288 bp long (**Fig. 1b**).

T7 endo I assay indicated high specific TALEN activity of 20 and 16%, for TAL 3 and TAL 6 respectively (**Fig. 1b**). We continued with TAL 3. To further determine the “indels” (insertions and deletions) generated by TAL 3 in C6 cells, we amplified the genomic DNA of the TAL 3-transfected C6 cells and performed a screening of the amplified *Nr3c1* gene. Screening results indicated a rate of 22.5% of NHEJ (Non Homologous End Joining, results not shown), which confirmed the results of the T7 endo I assay in cells. Cells transfected with TAL 3 presented mostly deletions ranging from 1 to 16 nucleotides (**Fig. 1c**).

### Donor molecule design and linearization

**Figure 2:**
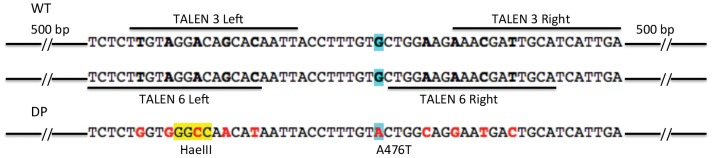
Donor plasmid design for the generation of GR^dim^ KI rat. Upper DNA indicates the wild type sequence of the exon 3 of the rat *Nr3c1* GR. In blue the residue A476. TAL 3 and TAL 6 binding sites are indicated. DP, donor plasmid sequence. Donor plasmid was synthesized with 500 pb homology arms on both ends. The pA476T point mutation is highlighted in blue. Silent mutations of the DP are shown in red bold letters and are located in the overlap of TAL 3 and Tal 6 binding sites. *Hae*III site is highlighted in yellow. *Alu*I site is not shown in this figure.

To generate GR^dim^ KI rats, we designed a common donor plasmid for TALENs 3 and 6, bearing the pA476T mutation along with 4 silent point mutations in each TALEN binding site to prevent further nuclease activity in the targeted alleles (**Fig. 2** **and Fig. S2 in File S1**). The donor plasmid sequence had also 500 bp homology arms on 3′ and 5′ sides of the pA476T mutation, to allow homology-derived recombination as described in [25]. The donor carried two extra point mutations for rapid detection by enzyme digestion: an *Alu*I site was removed and a *Hae*III site was added close to the pA476T site (**Fig. 2** **and Fig. S2 in File S1**).

### Generation of GR knockout and knockin rats by TALEN mRNA injection in one-cell stage embryos

TALEN mRNA and excised linearized double-stranded donor DNA containing point mutations and diagnostic restriction sites were co-injected into fertilized one-cell stage embryos at two concentrations (20 ng/µl or 10 ng/µl of each TALEN monomer mRNA) and at 5 ng/µl for donor DNA. As previously described [26] we performed a two-step microinjection procedure: we first injected the mixture TALEN/DNA into the male pronucleus and then into the cytoplasm during the withdrawal of the injection pipette. Data are summarized in **Table 1**.

**Table 1:** Injections of TAL 3 mRNA and donor plasmid DNA in rat one-cell embryos.

Dose TALEN mRNA/DNA (ng/µl)	No. Injected eggs/No. Transferred eggs (% of survival)	Offspring (% of injected eggs)	No. NHEJ pups (% of offspring)	No. KI pups (% of offspring)	Targeting frequency (% of offspring)
40 (20+20)/5	225/171 (76)	32 (14)	4 (13)	1 (3)	16
20 (10+10)/5	293/228 (79)	27† (9)	5 (19)	0	19

† One pup was born dead.

Two doses of TAL 3 mRNA were used (20+20 or 10+10 ng/µl of each TALEN). The egg survival rate is shown in percentage. NHEJ indicates the number of pups that had a gene disruption event in the sequence around pA476T. The percentages were calculated within each set of TALEN mRNA amount injected.

### Analysis of the HR events in Fo founders by *Alu*I and *Hae*III enzyme digestion and sequencing

**Figure 3:**
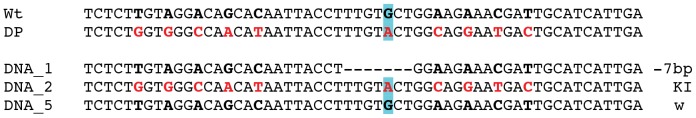
Founder KI female 3.4 genotyping from subcloned PCR amplicons of tail biopsies. Wt: Wild type; DP: donor plasmid. Point mutations in the DP are indicated in red bold letters. The pA476T mutation is highlighted in blue.

To reveal homologous recombination events located in the correct locus, we amplified the genomic DNA of the founders with the forward primer designed outside of the homology region and reverse primer inside of the homology region (“outside-in”) and digested them with *Alu*I and *Hae*III enzymes. Primers used are described in **Table S2 in File S1**. Gel digestion of the *Nr3c1* exon 3 revealed one KI female founder (namely 3.4) from the first injection series of higher TALEN mRNA out of 225 zygotes (KI founder gel digestion in **Fig. S3 in File S1**), and thus representing 1.7% of all live born pups. Sequencing of the *Nr3c1* exon 3 revealed that the 3.4 rat also presented a full donor DNA integration by homology-derived recombination (HDR). In addition to the KI allele, rat 3.4 also had a wild type and a 7 bp depleted alleles of the *Nr3c1* gene (**Fig. 3** **and Fig. S4 in File S1**).

### Analysis of the NHEJ events in Fo founders by *Alu*I enzyme digestion and sequencing

**Figure 4:**
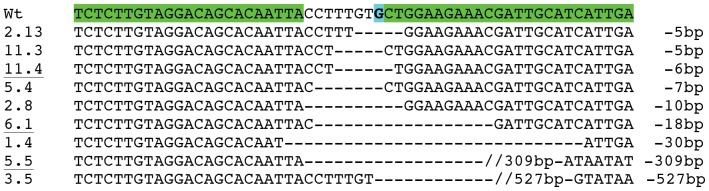
Fo KO rat genotyping. Wt, wild type sequence. TALEN binding sites are shown in green. The pA476T mutation is highlighted in blue. Longer deletions are marked with double slash. Rats 11.4, 6.1 and 5.5 (underlined) were kept for breeding. All rats beared the wt allele of the *Nr3c1* gene. Primers used are listed in **Table S2, File S1**.

To reveal NHEJ events in other founders, we digested the *Nr3c1* exon amplified with “inside-out” primers with *Alu*I enzyme. Nine NHEJ events were found resulting from both injection series (**Table 1** **and Fig. S5 in File S1**). Microinjection with each ratio TALEN/DNA resulted in both cases in high embryo survival (76% and 79% of injected embryos, respectively). All 9 founders showed *Nr3c1* deletions between 5 and 527 bp (data not shown). Three founder rats named 11.4, 6.1 and 5.5, were heterozygous and likely presented in-frame deletions of 6, 18 and 309 bp, respectively (**Fig. 4**). These mutations correspond to deletions of the dimerization and/or DNA-binding domain of the glucocorticoid receptor. We kept these 3 rats for further breeding.

**Figure 5:**
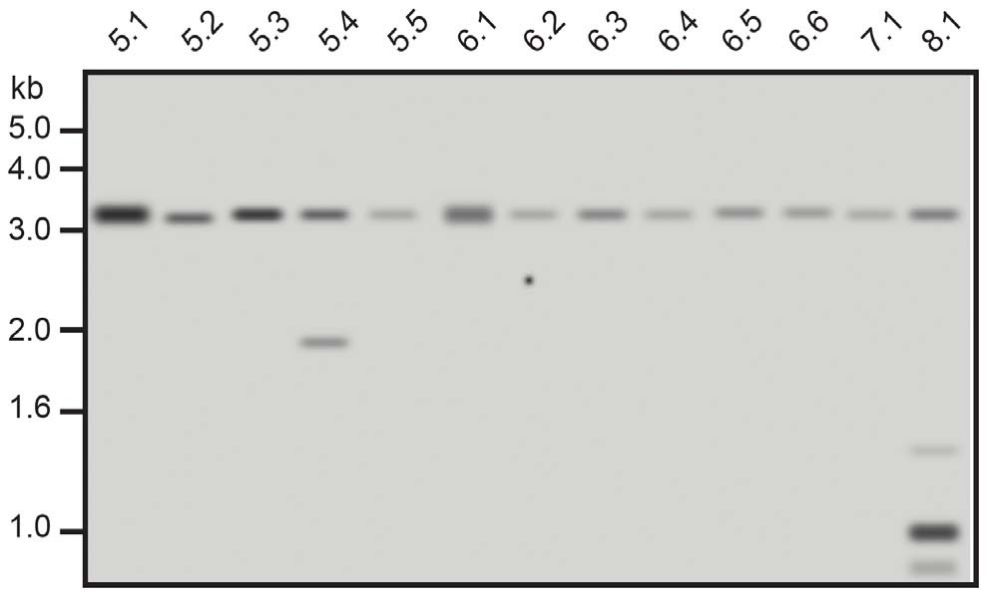
Detection of random donor integration in rat founders. Representative Southern blot analysis of founder rat genomic DNA following *Hin*cII digestion of genomic DNA and hybridization with exon 3-derived probe. Indicated is the 3.6 kb endogenous exon 3-containing genomic fragment and additional random integrations in founder 5.4 (knockout) and 8.1 (wildtype) rats. Rats 5.5 and 6.1 are knockouts as well but presented no off-target donor integration.

To check whether our linearized donor was integrated elsewhere into the genome, we performed a Southern blot analysis of all 58 founders using a hybridization probe around exon 3 and searched for additional integration events to the 3.6 kb *Hin*cII endogenous fragment (**Fig. 5**). In total, 7 live born pups out of 58 (12%) exhibited an off-target integration of the donor sequence. 5 out of 48 (10%) wild type founders and 2 out of 10 (20%) KO/KI founders harbored randomly one or several copies of the donor insert.

## Discussion

To our knowledge, here we report the first KI rat made by TALEN-mediated homology-derived recombination with a linear donor. In this study, we used linear donor since in previous injections, we were unable to obtain KI animals with a supercoiled donor. We observed 10 targeted mutations out of 58 pups born alive, representing 17% of the offspring. These results indicate high TALEN efficiency. Nine of the 10 *Nr3c1* gene modifications were knockouts and 1 was a knockin. Our single KI founder 3.4 harbored three *Nr3c1* alleles: KI (44%), KO (17%) and wt (39%), as analyzed by subcloning of the amplified *Nr3c1* exon 3 (results not shown). Offspring of the KI rat 3.4 died before giving rise to birth. NHEJ events were observed in 9 rats. Three analyzed and bred rats transmitted the mutated allele to the next generation confirming germline transmission. The 3 founders analyzed where heterozygous and had a deletion of several base pairs that are likely in-frame mutations of the glucocorticoid receptor, within the dimerization and/or DNA-binding domain. In 2 of the 3 founder lines, we obtained homozygous mutant offspring (Sofia Verouti, personal communication). They will be used for further physiological experiments.

Seven out of 58 live born founders (representing 12% of the offspring) presented off-site-target events. Of the 7 pups presenting off-target events, 2 had already on-target modifications. In some cases, off-targeted insertions were observed in up to three different loci in the same pup.

The rate of NHEJ found in rats was predicted efficiently by our assay in rat C6 cells and confirmed by screening (20% cutting efficiency determined by T7 endo I assay and 22.5% NHEJ by screening). Screening assays in cells indicated the generation of indels ranging from 1 to 16 bp, compared to deletions ranging from 5 to 527 bp in rats. 50% of the *Nr3c1*-targeted rats carried various *Nr3c1* alleles, including wild type sequences and deletions of several nucleotides.

This report serves as a proof of concept that TALENs are efficient tools to generate targeted and specific mutagenesis of the rat with linear donor molecules. They are affordable, convenient, freely designed and assembled in a molecular biology laboratory.

We demonstrate the first gene editing in the rat by homology-derived recombination (HDR) using oocyte microinjection of TALENs mRNA with a linear donor molecule. This report might encourage further TALEN-mediated gene targeting in the rat to apply these models in physiological and genetic research.

## Materials and Methods

### GR *Nr3c1* gene sequencing

The GR sequence around the pA476 site was sequenced from amplified genomic DNA pools of 10^6^ rat C6 cells (CCL-107, ATCC) and 6 adult Sprague-Dawley rats (Charles River Laboratory SAS SD rats): 3 males and 3 females. The primers used for the sequencing are listed in **Table S2 in File S1**.

### TALEN design and construction

TALEN pairs were designed using the free software from the website http://zifit.partners.org/ZiFiT/ and protocols for the standard cloning method and REAL assembly are available at http://www.talengineering.org/platforms-real.htm. The query sequence for TALEN design and the TALEN binding sites are available in **Table S1 in File S1**. TAL 3 was cloned by standard cloning according to instructions in http://zifit.partners.org/ZiFiT/Program_use.aspx#_TAL_Assembly. TAL 6 and 13 sequence were ordered for synthesis at Eurofins MWG Operon with 5′ overhang bearing the *Bbs*I site and the 3′ bearing the *Bsa*I site and then cloned into nuclease backbone vectors from the REAL protocol [12].

### Donor plasmid design

The donor plasmid had the pA476T site and homology arms made of 500 bp on the 3′ and 5′ ends. The *Alu*I site in intron 3 was suppressed and the silent mutation giving the *Hae*III site in exon 3 was added to test the insertion efficiency of the TALENs. Each TALEN binding site also carried 4 extra silent point mutations.

### Cell transfection, gene amplification and DNA preparation for the T7 endo I assay

10^5^ rat C6 cells were cultured in F-12 GlutamaX (Invitrogen, now Life Technologies), 10% FBS heat-inactivated and seeded in 6 well plates. Cells were transfected the next morning with plasmids encoding for TALENs at 0.6 µg total DNA per well and Lipofectamine 2000 (Invitrogen, now Life Technologies) following manufacturer's instructions. They were kept at 37°C for 4 hours in F-12 medium without FBS and Lipofectamine 2000. Then the medium was replaced by complete medium (F-12 and FBS), and cells were left for 72 hours at 30°C. Then cells were harvested and genomic DNA was extracted using Phire Animal Tissue Direct PCR Kit (Thermo Scientific) and amplified. Primers for amplification are listed in **Table S2 in File S1**. 5 µl of the PCR mix were heated at 95°C for 10 minutes and then the temperature was decreased by 5°C every minute until it reached 10°C in a Thermocycler.

### T7 Endo I mismatch detection assay and cloning

1 µl of NEB Buffer 2 (New England Biolabs) and, 0.5 µl of T7 Endo I nuclease (New England Biolabs) were added to the PCR mix previously prepared and incubated for 30 minutes at 37°C. Samples were loaded on agarose gel with 5 times concentrated loading buffer for analysis as described previously [26]. Amplified DNA was cloned into blunt vector using the TOPO cloning kit (Invitrogen, now Life Technologies) and transformed into HB101 bacteria. Colonies were seeded in 200 µl of LB medium in 96 well plates and sequenced.

### Animals

Sprague-Dawley rats (SD/Crl, Charles River) were housed in standard cages and protocols were conducted in accordance with the guidelines for animal experiments of the Veterinary Services and were performed by officially authorized personnel in a certified animal facility. All animal experiments were compliant with the Animal Protection Law of the French republic (article R-214-89), which is in compliance with the European Community Council recommendations for the use of laboratory animals 86/609/ECC, and were approved by the CEEA Pays de la Loire committee (ref: CEEA-2011-45).

### Cell lysis and Western blot analysis

C6 cells were harvested and lysed with 1% Triton buffer (1 M Tris, 5 M NaCl, 1% Triton X-100 (Sigma), 1∶500 vol/vol of the following: leupeptin, aprotinin and pepstasin (Sigma) and 1∶1000 vol/vol of PMSF (Sigma)) for 30 minutes at 4°C in a roller. Then cell lysis was centrifuged 15 minutes at 15000 rpm to remove cell membrane debris. The supernatant containing the proteins was loaded on a 10% acrylamide gel with denaturing sample buffer after incubated at 95°C for 5 minutes. Proteins were transferred to a nitrocellulose membrane and blocked with 5% milk TBS. After washing, the membrane was incubated with 1∶1000 monoclonal antibody against flag protein produced in mouse (Sigma) and secondary antibody was anti-mouse IgG linked to horseradish peroxidase (GE Healthcare). The film was exposed 5 minutes.

### In vitro transcription of TALEN mRNA

For the pA476T gene targeting, TALEN-encoding expression plasmids were linearized with *Pme*I. Messenger RNA was in vitro transcribed and polyadenylated using the mMessage mMachine T7 Ml ultra kit (Ambion) following the manufacturer protocol and purified using the MegaClear Kit (Ambion), quantified using a NanoDrop-1000 (Thermo Scientific) and stored at −80°C until use. Messenger RNAs encoding pA476T TALENs were mixed to a final total concentration of 10 ng/µl, or 20 ng/µl of each TALEN monomer in TE 5/0.1 (5 mM Tris-Cl pH 7.5, 0.1 mM EDTA in RNase DNase free water) and stored at −80°C until use. mRNAs were kept on ice during all micro-injection procedures.

### Linearization of donor DNA

Donor plasmid for GR^dim^ TAL 3 and TAL 6 was digested with *Not*I to excise the donor DNA from the vector backbone for HDR. After electrophoresis, digested linear donor DNA was cut from agarose gel, electroeluted and purified with Elutip-d column (Whatman). Linear DNA was quantified using a NanoDrop-1000 and stored at −20°C until use. Linear donor DNA was mixed at 5 ng/µl with TALENs mRNA and stored at −80°C until use.

### Microinjection into rat zygotes

Prepubescent females (4–5 weeks old) were injected with 30 IU pregnant mare serum gonadotropin (Intervet) and followed 48 hours later with 20 IU human chorionic gonadotropin (Intervet) before breeding as previously described [27]. Fertilized one-cell stage embryos were collected for subsequent microinjection using a previously published procedure [27]. Briefly, a mixture of TALEN mRNA and donor DNA was microinjected both into the male pronucleus and into the cytoplasm of fertilized one-cell stage embryos. Two ratios of diluted TALEN mRNA and donor DNA have been tested. Surviving embryos were implanted on the same day in the oviduct of pseudo-pregnant females (0.5 dpc) and allowed to develop to full term.

### Rat genotyping experiments

DNA from neonates was extracted from tail biopsy following treatment with Proteinase K. Rat tail biopsies where incubated overnight at 55°C in 500 µl of Proteinase K solution (0.2 M NaCl, 1.1 M Tris (pH 8.3), SDS 0.2%, EDTA 5 mM and 100 µg/ml proteinase K (Sigma)). DNA was extracted following NaCl method by adding 6 M of NaCl, mixing and quick spin down. Supernatant was mixed with 2/3 of the volume of isopropanol, and vortexed for 2 minutes. Solution was centrifuged at 10000 rpm and the supernatant was removed. DNA pellet was washed with 1.5 ml of 70% ethanol and then resuspended in bi-distilled water for 2 hours at 37°C. Genotyping and sequencing was performed using primers listed in **Table S2 in File S1**. Amplicons were digested with either *Hae*III or *Alu*I enzymes then ran on a 1.8% agarose gel.

### Southern blot

Southern blot analysis of founders following *Hin*cII digestion of tail DNA and hybridization with a duplicated PCR-amplified exon 3 probe was performed using standard procedures. Briefly, the exon 3 sequence was amplified by PCR using primers listed in **Table S2 in File S1** inserted in 2 concatemers into TOPO cloning vector and isolated as an *Eco*RI fragment for hybridization using standard conditions.

## Supporting Information

File S1 **Supplementary Data are available at PLoS ONE Online and include Table S1–S2 and Figure S1–S5.** Table S1: *Nr3c1* sequence for TALEN design and binding site sequences. Table S2: Primers used in this experiment. Figure S1 Evaluation of TALEN expression in C6 rat cells. C6 cells were transfected with either TAL 3, 6 or 13, Right (R), Left (L) or both (RL). Western blot was performed with antibody against flag tag. Mcells are mock-transfected cells. Figure S2 Sequence of the *Nr3c1* gene donor plasmid. The homology arms are indicated in purple, the exons and introns of the *Nr3c1* gene are indicated in orange and black lines respectively. TALEN binding sites are indicated in blue and the spacer in red. Nucleotides that are mutated in the donor are marked in bold letters. *Hae*III site in the donor is underlined in blue. Suppressed *Alu*I site (is underlined in purple). Figure S3 Gel digestion analysis of the 3.4 rat *Nr3c1* exon 3. Primer pairs used in this experiment (“outside-in”) are shown in Table S2 in File S1. Wt indicates expected fragments of wild type animals for both *Alu*I and *Hae*III enzymes. The stars indicate bands expected of the donor sequence: for *Alu*I digestion: one upper band of 883 bp (*), for *Hae*III digestion: two bands of 524 (**) and 359 bp (***). Rat 3.4 also shows wt digestion pattern, indicating that it is heterozygous for the pA476T mutation. Figure S4 Sequencing of the *Nr3c1* gene in 3.4 KI rat. Genomic DNA was amplified with primers outside out the donor sequence (“outside-out”) (c. f. Table S2 in File S1), and cloned into TOPO cloning vector. 21 clones were selected for sequencing. Here we show 3 representative sequences. Wt, wild type sequence; exp, expected sequence, DNA1, clone 1, DNA2, clone 2, DNA5, clone 5. TALEN binding sites from the donor are in green, bold letters indicate the mutated nucleotides. *Hae*III site is present in the donor sequence only; *Alu*I is present in the wild type sequence only. Figure S5 Gel digestion analysis of “indels” in Fo founders rats. “Inside-out” primers were used in this assay (Table S2 in File S1). Wt indicates expected fragments of wild type animals upon digestion with *Alu*I enzyme. All pups have variable size bands on the lower molecular weight zone, indicating possible indels. The KI rat 3.4 upper band (marked with a * of 469 bp) indicates absence of the *Alu*I site close to the pA476T. KI fragment sizes expected: 125, 250 and 469 bp Wt fragment sizes expected: 125, 175, 250 and 294 bp.
(DOCX)
