# Supplementary material for: Generation of TALEN-Mediated GRdim Knock-In Rats by Homologous Recombination
Source: PLoS One. 2014 Feb 11;9(2):e88146. doi: 10.1371/journal.pone.0088146 (PMC3921256; doi:10.1371/journal.pone.0088146)
Supplement: File S1 — Supplementary Data are available at PLoS ONE Online and include Table S1–S2 and Figure S1–S5. Table S1: Nr3c1 sequence for TALEN design and binding site sequences. Table S2: Primers used in this experiment. Figure S1 Evaluation of TALEN expression in C6 rat cells. C6 cells were transfected with either TAL 3, 6 or 13, Right (R), Left (L) or both (RL). Western blot was performed with antibody against flag tag. Mcells are mock-transfected cells. Figure S2 Sequence of the Nr3c1 gene donor plasmid. The homology arms are indicated in purple, the exons and introns of the Nr3c1 gene are indicated in orange and black lines respectively. TALEN binding sites are indicated in blue and the spacer in red. Nucleotides that are mutated in the donor are marked in bold letters. HaeIII site in the donor is underlined in blue. Suppressed AluI site (is underlined in purple). Figure S3 Gel digestion analysis of the 3.4 rat Nr3c1 exon 3. Primer pairs used in this experiment (“outside-in”) are shown in Table S2 in File S1. Wt indicates expected fragments of wild type animals for both AluI and HaeIII enzymes. The stars indicate bands expected of the donor sequence: for AluI digestion: one upper band of 883 bp (*), for HaeIII digestion: two bands of 524 (**) and 359 bp (***). Rat 3.4 also shows wt digestion pattern, indicating that it is heterozygous for the pA476T mutation. Figure S4 Sequencing of the Nr3c1 gene in 3.4 KI rat. Genomic DNA was amplified with primers outside out the donor sequence (“outside-out”) (c. f. Table S2 in File S1), and cloned into TOPO cloning vector. 21 clones were selected for sequencing. Here we show 3 representative sequences. Wt, wild type sequence; exp, expected sequence, DNA1, clone 1, DNA2, clone 2, DNA5, clone 5. TALEN binding sites from the donor are in green, bold letters indicate the mutated nucleotides. HaeIII site is present in the donor sequence only; AluI is present in the wild type sequence only. Figure S5 Gel digestion analysis of “indels” in Fo fo [file pone.0088146.s001.docx]

PLoS ONE

S1: Supporting information for

**Generation of TALEN-mediated GR^dim^ knockin rats by homologous recombination**

Verónica Ponce de León, Anne-Marie Mérillat, Laurent Tesson, Ignacio Anegón, Edith Hummler

| **Supplementary Item** | **Title of Caption** |
| --- | --- |
| **Supplementary Table 1** | *Nr3c1* sequence for TALEN design and binding site design |
| **Supplementary Figure 1** | Western blot of the TALEN expression in C6 cells |
| **Supplementary Table 2** | Primers used in this experiment |
| **Supplementary Figure 2** | Donor plasmid sequence |
| **Supplementary Figure 3** | Gel digestion analysis of the *Nr3c1* exon 3 of the 3.4 rat. |
| **Supplementary Figure 4** | Sequencing results of the 3.4 rat *Nr3c1* gene |
| **Supplementary Figure 5** | Gel digestion analysis of Fo founder rats. |

***Ponce de León et al,***

**Table S1: *Nr3c1* sequence for TALEN design and binding site sequences**

| **Item name** | **Sequence of the item** |
| --- | --- |
| ***Nr3c1* query sequence for all 18 TALENs** | 5’-CCAGAAAGAGGATAGGCTTTTCATTAAAAATGTCCTTACAGGTCTGTGGAATTTTA  ACAATGCCCTTTACTCTCTTGTAGGACAGCACAATTACCTTTGT[G]CTGGAAGAAACGATTGCATCATTGATAAAATTCGAAGGAAAAACTGCCCAGCATGCCGCTATCGGAAATGTCTTCAGGCTGGAATGAACCTTGAAGGTA-3’ |
| **TAL 3 binding sites** | 5**’-TTGTAGGACAGCACAATT**AACCTTTGTGCTGGAAG**AAACGATTGCATCATTGA-**3’ |
| **TAL 6 binding sites** | 5’-**TCTCTTGTAGGACAGAC**AATTACCTTTGTG**CTGGAAGAAACGATTGCA-**3’ |
| ***Nr3c1* query sequence for TALEN 13** | 5’-CCAGAAAGAGGATAGGCTTTTCATTAAAAATGTCCTTACAGGTCTGTGGAATTTTA  ACAATGCCCTTTACTCTCTTGTAGGACAGCACAATTACCTTTGTG[C]TGGAAGAAACGATTGCATCATTGATAAAATTCGAAGGAAAAACTGCCCAGCATGCCGCTATCGGAAATGTCTTCAGGCTGGAATGAACCTTGAAGGTA-3’ |
| **TAL 13 binding sites** | 5’-**TCTTGTAGGACAGCACAA**TTACCTTTGTGCTGGAAGAAACG**ATTGCATCATTGA**  **TAAAA-**3’ |

**Figure S1 : Evaluation of TALEN expression in C6 rat cells**


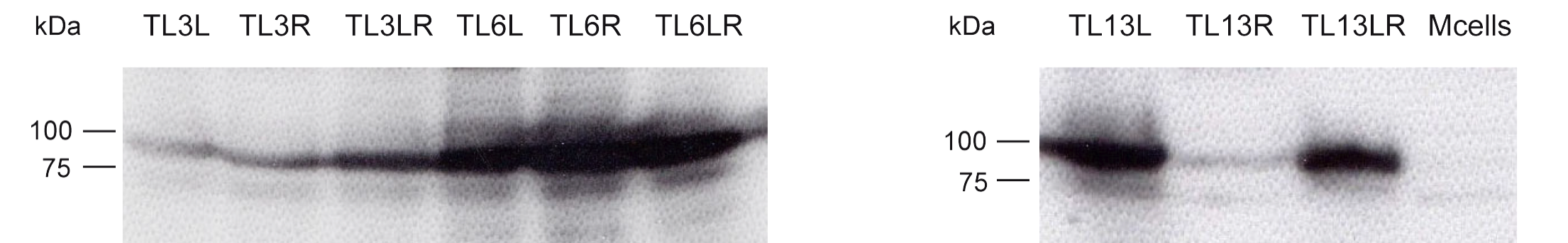


***Ponce de León et al,***

**Table S2: Primers used in this experiment**

| **Template DNA** | **Sequence of the primer** |
| --- | --- |
| ***Nr3c1*  gene sequencing** | F: 5’-GCTGGAAGTTGGTTCATTT-3’  R: 5’-TCAATTCCTTAGTTCCTCAATATAAG -3’ |
| **T7 endo 1 assay** | F: 5’-CTCTCAACATGGTAATTCATGTAGAAAAG-3’  R: 5’-AATATACACCCTGGCTGTCC -3’ |
| **« Outside-in» *Nr3c1* primers** | F : 5‘-GCTGGAAGTTGGTTCATTT-3’  R : 5’-CAAAATTGCTCCAAGTTACG-3’ |
| **« Inside-out » *Nr3c1* primers** | F : 5‘-GTCTGTGGAATTTTAACAATGC-3’  R : 5’-GCTGCTCAGACTCAGGCAC-3’ |
| **«Outside-out » *Nr3c1* primers** | F : 5’-GCTGGAAGTTGGTTCATTT-3’  R : 5’-CCCTGGAACTAAGTTACTGGC-3’ |
| **Fo founder sequencing** | F: 5’-GCTGGAAGTTGGTTCATTT-3’  R: 5’-CGTAACTTGGAGCAATTTTG-3’ |
| **DNA probe for Southern blot analysis** | F: 5’-GTGTCGAATTCCTTCAAGGTTCATTCCAG-3’  R: 5’-GTGTCGAATTCGACAGCACAATTACCTTTG-3’ |

***Ponce de León et al,***

**Figure S2 : Sequence of the *Nr3c1* gene donor plasmid**

***Ponce de León et al,***

**Figure S3 : Gel digestion analysis of the 3.4 rat *Nr3c1* exon 3.**

**^
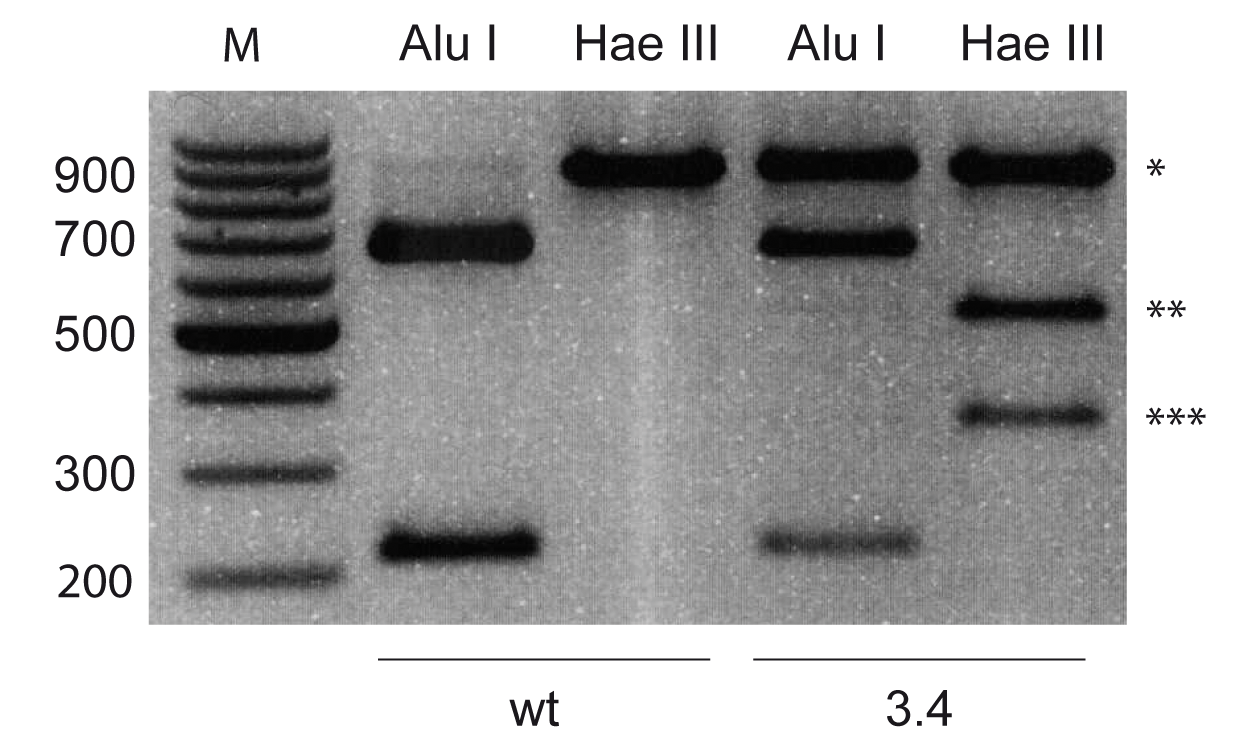
^**

***Ponce de León et al,***

**Figure S4 : Sequencing of the *Nr3c1* gene in 3.4 KI rat**

**^^**

***Ponce de León et al,***

**Figure S5 : Gel digestion analysis of « indels » in Fo founder rats**

**^
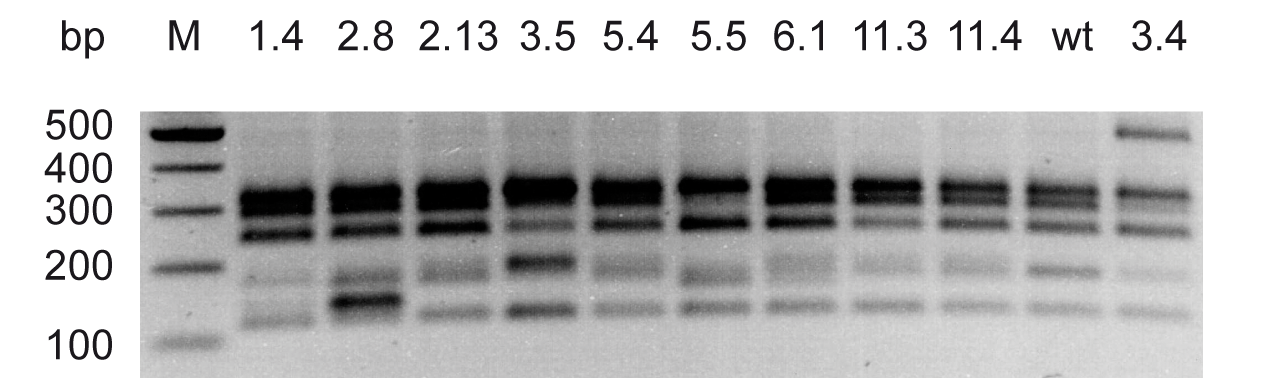
^**

*
